# Switching from originator infliximab to biosimilar infliximab in Japanese patients with rheumatoid arthritis achieving clinical remission (the IFX-SIRIUS study I): Study protocol for an interventional, multicenter, open-label, single-arm and noninferiority clinical trial with clinical, ultrasound, and biomarker assessments

**DOI:** 10.1097/MD.0000000000021151

**Published:** 2020-07-24

**Authors:** Shin-ya Kawashiri, Toshimasa Shimizu, Shuntaro Sato, Shimpei Morimoto, Yurika Kawazoe, Remi Sumiyoshi, Naoki Hosogaya, Chizu Fukushima, Hiroshi Yamamoto, Atsushi Kawakami

**Affiliations:** a Departments of^1^Community Medicine; b Immunology and Rheumatology, Division of Advanced Preventive Medical Sciences, Nagasaki University Graduate School of Biomedical Sciences; c Clinical Research Center, Nagasaki University Hospital; d Innovation Platform & Office for Precision Medicine, Nagasaki University Graduate School of Biomedical Sciences, Nagasaki, Japan.

**Keywords:** biomarker, biosimilar, CT-P13, infliximab, musculoskeletal ultrasound, originator, rheumatoid arthritis

## Abstract

**Background:**

The introduction of biological disease-modifying anti-rheumatic drugs (bDMARDs) into clinical practice has dramatically improve the clinical outcomes of individuals with rheumatoid arthritis (RA). However, bDMARDs are associated with high costs, which has resulted in restricted treatment access and a burden on medical insurance finances. Although biosimilars offer cost-saving, their effectiveness and safety must be established in Post-Marketing Surveillance (PMS). Infliximab (IFX), a chimeric monoclonal antibody to TNF-alpha, is the first bDMARD; its biosimilar, CT-P13, is the first biosimilar DMARD approved for RA treatment in Japan. We will evaluate whether switching from originator IFX to CT-P13 is not inferior for maintaining non-clinical relapse to continued treatment with originator IFX in RA patients achieving clinical remission.

**Methods/design:**

This study is an interventional, multicenter, open-label, single-arm against historical control and noninferiority clinical trial with a 24-week follow-up. Eighty RA patients who are treated by originator IFX for ≥24 weeks and are achieving clinical remission will be included. Patients will be switched to CT-P13 with the unchanged dosing regimen. We will evaluate disease activity by measuring clinical disease activity indices and by using musculoskeletal ultrasound (MSUS). The primary endpoint is the ratio of patients who experience a nonclinical relapse during the study period. Important secondary endpoints are the changes from the baseline of the MSUS scores. We will also comprehensively analyze the serum levels of many biomarkers such as cytokines and chemokines.

**Discussion:**

The study results are expected to show the noninferiority of switching to CT-P13 over the continuation of originator IFX. The strength of this study is its prospective evaluation of therapeutic efficacy using not only clinical disease activity indices but also MSUS to accurately and objectively evaluate disease activity at the joint level among patients drawn from multiple centers with a standardized evaluation by MSUS. We will explore whether parameters at baseline can predict a nonclinical relapse after switching from originator IFX to CT-P13 by integrating multilateral assessments, i.e., clinical disease activity indices, MSUS findings, and serum biomarkers.

**Trial registration:**

This study was registered in the Japan Registry of Clinical Trials (https://jrct.niph.go.jp) on October 11, 2019 as jRCTs071190030.

## 1 Introduction

Rheumatoid arthritis (RA) is characterized by persistent synovitis, systemic inflammation, and autoantibodies.^[1]^ Uncontrolled active RA causes joint damage, disability, decreased quality of life, and comorbidities. The tight control of the disease activity of RA following the treat-to-target (T2T) strategy is thus recommended.^[2]^ Advances in the treatment of RA, such as the use of biological disease-modifying anti-rheumatic drugs (bDMARDs), have provided better clinical outcomes, including the achievement of clinical remission for patients with RA. Clinicians also aim to achieve not only clinical remission but also imaging remission and immunological remission.^[3]^

The pathophysiology of RA is associated with several inflammation cascades. One key inflammation cascade includes the overproduction and overexpression of tumor necrosis factor (TNF). This pathway drives both synovial inflammation and joint destruction.^[1]^ Infliximab, a chimeric monoclonal antibody to TNF-alpha, was the first bDMARD to demonstrate a dramatic change in the treatment of RA. IFX is extremely effective in suppressing disease activity and the progression of joint destruction.^[4–6]^ However, although bDMARDs are highly effective, they are costly.

CT-P13 is a biosimilar of originator IFX, developed by Celltrion (Incheon, South Korea). A “biosimilar” is a biotherapeutic product that is similar in terms of quality, safety, and efficacy to an already licensed reference biotherapeutic product (i.e., originator).^[7]^ CT-P13 was approved in 2014 as the first biosimilar DMARD (bsDMARD) for RA treatment in Japan.^[8]^ The introduction of bsDMARDs is expected to reduce the patients economic burden and improve medical insurance finances. The biosimilar CT-P13 and the originator IFX have been shown to be pharmacokinetically equivalent and comparable in efficacy and safety.^[8]^ Switching from originator IFX to CT-P13 was reported to be not clinically inferior to continued treatment with originator IFX^[9]^ or CT-P13.^[10]^ However, in all of these previous studies, the endpoints of efficacy were based on clinical disease activity indices, and the evaluation of disease activity based on high-sensitivity imaging modalities such as joint musculoskeletal ultrasound (MSUS) was not performed.

MSUS is usually used to evaluate the disease activity of RA,^[11,12]^ and MSUS experts have stated that RA patients treated with DMARDs should undergo assessments with MSUS since MSUS better shows the activity of synovial inflammation compared to a clinical examination,^[11,12]^ indicating that the use of MSUS to assess the therapeutic response can be of great help in clinical practice.^[11–14]^ MSUS is a noninvasive, objective, relatively inexpensive, and repeatable imaging modality that is suitable for treatment monitoring.^[11,12]^

As mentioned above, clinical remission can be achieved in a relatively large number of RA patients by introducing bDMARD therapy, but residual synovitis detected by MSUS is known to remain at a certain frequency even in patients who achieve clinical remission.^[15,16]^ The residual synovitis is an important finding that predicts joint destruction and clinical relapse. It is therefore important to accurately evaluate disease activity at the joint level by using MSUS as well as clinical disease activity indices including subjective parameters. We will therefore also use MSUS assessments to determine whether switching from originator IFX to CT-P13 is not inferior to continued treatment with originator IFX in RA patients achieving clinical remission. A multicenter collaborative study that prospectively evaluates disease activity using MSUS standardized at a high level is rare, even worldwide.

We will evaluate the changes of disease activity after RA patients are switched from originator IFX to CT-P13 by using MSUS as well as clinical disease activity indices so that the patients disease activity will be more accurately assessed. We will also comprehensively analyze the serum level of many biomarkers such as cytokines and chemokines. We will explore whether the patients parameters at baseline can be used to predict a nonclinical relapse after switching from originator IFX to CT-P13 by integrating multilateral assessments including clinical disease activity indices, MSUS findings, and biomarkers.

We named this clinical trial Infliximab Similarity Investigation by Ultrasound Study I (IFX-SIRIUS STUDY I). Herein, we describe the final protocol (version 2.0; December 15, 2019) for the study. We are also planning a study (IFX-SIRIUS STUDY II) to evaluate the changes in disease activity by MSUS as well as clinical disease activity indices after the discontinuation of CT-P13 in patients who have not experienced a clinical relapse following this study.

## 2 Objectives

### 2.1 Primary objective

The principal objective of the study is to determine whether switching from originator IFX to CT-P13 is not inferior in maintaining nonclinical relapse to continued treatment with originator IFX in RA patients achieving clinical remission.

### 2.2 Secondary objectives

We will assess disease activity by MSUS after patients are switched from originator IFX to CT-P13. We will explore whether the clinical and/or MSUS findings and/or biomarkers at baseline can be used to predict a nonclinical relapse after switching from originator IFX to CT-P13.

## 3 Methods/design

### 3.1 Study design

**Figure 1:**
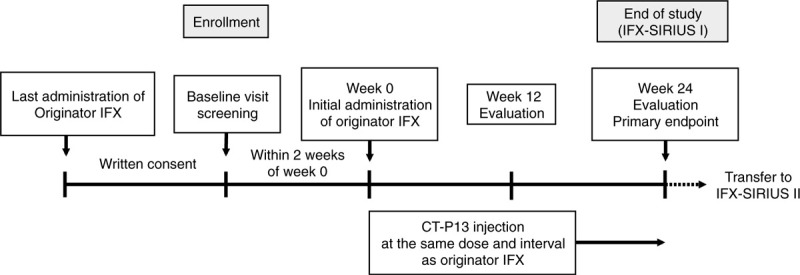
Study design. IFX = infliximab.

The study design is in accordance with the Standard Protocol Items: Recommendations for Interventional Trials and Consolidated Standards of Reporting Trials 2010 guidelines.^[17,18]^ (Additional File 1). The study is a prospective, open-label, interventional and noninferiority clinical trial. This is a single-arm against historical control using a previous report^[19]^ and a multicenter RA ultrasound prospective cohort we are investigating.^[20]^ The study will be conducted at the following 17 centers: Nagasaki University Hospital, Asahi General Hospital, National Hospital Organization Chiba-East Hospital, Eiraku Clinic, Hamanomachi Hospital, Japanese Red Cross Nagasaki Genbaku Hospital, Kagawa University Hospital, University of Miyazaki Hospital, Nagasaki Kita Hospital, Osaka City University Hospital, Osaka Medical College Hospital, Sagawa Akira Rheumatology Clinic, Sasebo Chuo Hospital, Sasebo City General Hospital, Shiminnomori Hospital, Utazu Hospital, and Yoshitama Clinic for Rheumatic Diseases. In total, 80 patients with RA will be assigned to switch from originator IFX to CT-P13. The duration of the intervention is 24 weeks. The study design is summarized in Figure 1.

### 3.2 Approvals

The study was approved by the certified review board (CRB) of Nagasaki University (CRB approval no.: CRB19-010). The study is registered in the Japan Registry of Clinical Trials (https://jrct.niph.go.jp) as jRCTs071190030. We will conduct the study in accordance with the principles of the Declaration of Helsinki and the Clinical Trials Act (Act No. 16 of April 14, 2017), the Act on the Protection of Personal Information and related regulatory notifications, and this clinical study protocol. Participants will be provided with an explanation regarding the study by their treating rheumatologist and will be asked to voluntarily sign an informed consent form before their participation.

### 3.3 Participants

#### 3.3.1 Inclusion criteria

Patients must meet all of the following requirements to be considered for entry into the study:

1. ≥20 years old,
2. with the diagnosis of RA based on the American College of Rheumatology (ACR) /European League Against Rheumatism (EULAR) 2010 RA Classification Criteria,^[21]^
3. treated with originator infliximab for ≥24 weeks and achieving clinical remission defined as a Disease Activity Score 28 (DAS28)-erythrocyte sedimentation rate (ESR) <2.6 at the last administration of originator IFX and at baseline,
4. able and willing to give written informed consent and comply with the requirements of the study protocol.

#### 3.3.2 Exclusion criteria

The exclusion criteria are as follows:

1. the concurrent use of a corticosteroid equivalent to >10 mg/day of prednisolone,
2. a previous use of a biosimilar for IFX,
3. treated with a biologic DMARD other than originator IFX or a bsDMARD,
4. a history of infusion reaction due to originator IFX that required medication,
5. treated with a corticosteroid or antirheumatic drug and changed the dose within 8 weeks prior to the baseline visit,
6. the use of a prohibited drug or therapy within 8 weeks prior to the baseline visit,
7. current pregnancy, breastfeeding, or noncompliant with a medically approved contraceptive regimen during and 6 months after the study period, or
8. being considered unsuitable for this study by the investigator.

#### 3.3.3 Sample size

The sample size with a statistical power of 0.8, in a test for noninferiority with a significance level of 0.05, was estimated based on the results from the test for noninferiority on 2000 simulation datasets. Each simulation dataset was generated by random sampling from a binomial distribution with a probability of 0.16. The reason for the probability (0.16) and the procedures in the test for noninferiority are detailed below in Section 3.8.

#### 3.3.4 Intervention

Patients will receive intravenous CT-P13 with an unchanged dosing regimen throughout the study period. CT-P13 will be administered at the same dose (dose per kg) and the same interval as the originator IFX before switching.

All of the patients must continue to take the same doses of methotrexate (MTX) and oral corticosteroid that they were taking before the switch, throughout the study period. During the study period, the following treatments are prohibited: the administration of a bDMARD or JAK inhibitor, the concomitant use of an immunosuppressant (azathioprine, cyclophosphamide, cyclosporine) or oral corticosteroids equivalent to >10 mg/day of prednisolone, intra-articular corticosteroid injections at joints, and nonsteroidal anti-inflammatory drug (NSAID) suppositories. During the study period, the dosage of any NSAID can be modified within the range of its approved doses in Japan.

#### 3.3.5 Patient discontinuation criteria

A patient may be discontinued prematurely for the following reasons (patients who discontinue before completing the trial will not be replaced):

- Patient experiences a clinical relapse.
- CT-P13 must be reduced or discontinued for some reason(s).
- Continuing participation is inadvisable due to adverse event(s).
- The patient asks to leave the trial.
- In the Principal Investigator's opinion, continuation in the trial would be detrimental to the patient's well-being.

### 3.4 Outcome measurements

Study visits will take place at baseline and after 12 and 24 weeks of treatment. The assessments are presented in Table 1. Clinical assessors will be blinded to joint assessments by MSUS.

**Table 1:**
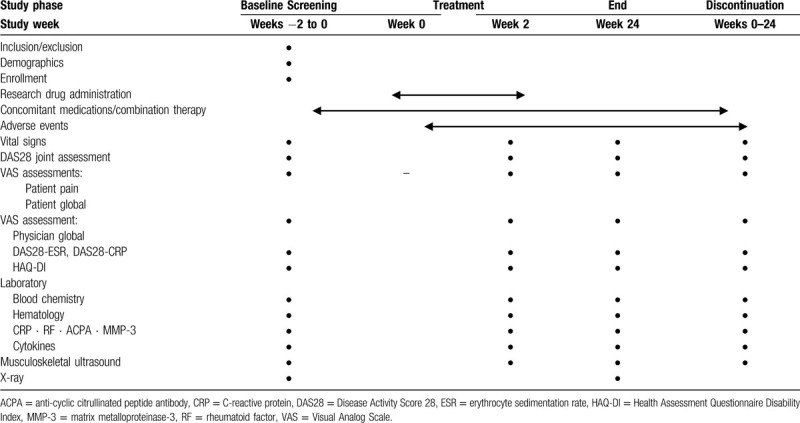
Treatment schedule and outcome measures.

#### 3.4.1 Clinical disease activity

Clinical disease activity was evaluated by each of the attending physicians (Japan College of Rheumatology [JCR]-certified rheumatologists) based on the values of the DAS28-ESR and the DAS28-C reactive protein (CRP) level.^[22]^ At each visit, 28 joints including the bilateral glenohumeral, elbow, wrist, metacarpophalangeal (MCP), interphalangeal (IP), proximal interphalangeal (PIP) of the hand, and knee joints were assessed for tenderness and swelling. Each patients global assessment (PtGA) and evaluators global assessment (EGA) will be established on a 0–100 mm visual analog scale. The patients’ functional assessment will be evaluated by the Health Assessment Questionnaire-Disability Index (HAQ-DI).^[23]^

#### 3.4.2 MSUS assessments

Participants will undergo imaging by MSUS at baseline, week 12 and week 24. The MSUS examination of each patient will be performed by 1 of the JCR-certified sonographers. A systematic multiplanar grayscale (GS) and power Doppler (PD) examination of each patient's joints will be performed using a multifrequency linear transducer (12–24 MHz). PD will be used depending on which Doppler modality is the most sensitive on the individual machines. The Doppler settings will be adjusted at each hospital according to published recommendations.^[24]^ There will be no change in the MSUS settings during the study and no upgrading of software.

Articular synovitis will be assessed by MSUS at dorsal views of 22 joints: bilateral wrist joints, 1st–5th MCP joints, the IP joints, and the 2nd–5th PIP joints. Each joint is scored for GS as well as PD on a scale from 0 to 3 in a semiquantitative manner. The sum of the GS scores or the PD scores is considered the total GS score or total PD score, respectively. We will also assess the Outcome Measures in Rheumatology (OMERACT)-EULAR combined PDUS score (i.e., the combined PD score) and the Global OMERACT-EULAR Synovitis Score (GLOESS).^[25,26]^ The combined PD score is combined with synovial hypertrophy shown by GS and PD.^[25,26]^

#### 3.4.3 X-ray imaging

X-ray images of bilateral hands (posteroanterior view) and feet (anteroposterior view) will be conducted. Joint damage progression will be evaluated based on the van der Heijde-modified total Sharp score (vdH-mTSS) method including 16 areas in each hand for erosions and 15 for joint-space narrowing.^[27]^

#### 3.4.4 Biomarker measurements

The patients serum concentrations of the following biomarkers will be measured. Rheumatoid factor (RF) will be measured by a latex agglutination turbidimetric immunoassay (LATIA) (LZ test “Eiken” RF). Anti-cyclic citrullinated peptide antibodies (ACPA) will be measured by a chemiluminescent immunoassay (CLEIA) (STACIA MEBLux test CCP). Matrix metalloproteinase-3 (MMP-3) will be measured by a latex turbidimetric immunoassay (LTIA) (Panaclear MMP-3 ’Latex’). Multiplex cytokine/chemokine bead assays will be performed using diluted serum supernatants and a MILLIPLEX MAP Human Cytokine/Chemokine Magnetic Bead Panel (Merck Millipore) – Bio-Plex Pro Human Cytokine Assays (Bio-Rad) analyzed with a Bio-Plex MAGPIX Multiplex Reader (Bio-Rad) according to the manufacturer's instructions.

The cytokines/chemokines that are measured by the bead panel include interleukin (IL)-1α, IL-1β, IL-1 receptor antagonist, IL-2, IL-3, IL-4, IL-5, IL-6, IL-7, IL-8, IL-9, IL-10, IL-12 (p40), IL-12 (p70), IL-13, IL-15, IL-17, IL-18, interferon-gamma (IFN-γ), IFN-α2, CXCL1 (growth-related oncogene [GRO]), granulocyte-macrophage colony-stimulating factor (GM-CSF), granulocyte colony-stimulating factor (G-CSF), CX3CL1 (fractalkine), flt-3 ligand, fibroblast growth factor (FGF)-2, eotaxin, epidermal growth factor (EGF), soluble CD40 ligand (sCD40L), vascular endothelial growth factor (VEGF), TNF-β, TNF-α, transforming growth factor (TGF)-α, CCL4 (macrophage inflammatory protein [MIP]-1β), CCL3 (MIP-1α), CCL22 (macrophage-derived chemokine [MDC]), CCL7 (monocyte chemotactic protein [MCP]-3), CCL2 (MCP-1), CXCL10 (IFN-γ-inducible protein [IP]-10), vascular cell adhesion molecule-1 (VCAM-1), and intercellular adhesion molecule-1 (ICAM-1). The serum levels of IL-6 and TNFα will be measured with specific enzyme-linked immunosorbent assay (ELISA) kits (R&D Systems).

### 3.5 Study endpoints

#### 3.5.1 Primary endpoint

The primary endpoint is the proportion of patients who experience a clinical relapse during the period from baseline to week 24. Clinical relapse is defined as

1. a change from the baseline value in the DAS28-ESR (ΔDAS28-ESR) ≥1.2 or in the DAS28-ESR ≥3.2, and
2. an increase in the DAS28-ESR value due to elevated disease activity of RA.

#### 3.5.2 Secondary endpoints

The secondary endpoints of this study are the following:

1. the changes of the total PD and GS scores and the combined PD score from baseline to weeks 12 and 24, and
2. the changes in the DAS28-ESR and DAS28-CRP values from baseline to weeks 12 and 24.

#### 3.5.3 Exploratory endpoints

For further research we will assess the following:

1. the change in vdH-mTSS from baseline to week 24
2. the change in the HAQ-DI data from baseline to weeks 12 and 24
3. the changes in the serum levels of biomarkers from baseline to weeks 12 and 24
4. the DAS28-ESR and DAS28-CRP values at baseline and weeks 12 and 24
5. the total PD and GS scores and the combined PD score at baseline, week 12, and week 24
6. the vdH-mTSS at baseline and week 24
7. the HAQ-DI at baseline, week 12, and week 24
8. the serum levels of biomarkers at baseline, week 12, and week 24

### 3.6 Adverse events

All adverse events (AEs) that occur between the administration of CT-P13 and the end of week 24 will be recorded. If necessary, the investigators will administer treatments. A serious AE (SAE) is defined as any adverse reaction resulting in any of the following outcomes: a life-threatening condition or death; a condition that requires inpatient hospitalization or the prolongation of existing hospitalization; threatening to cause disability or disability, a congenital anomaly, or a birth defect. All SAEs will be documented in the medical records and reported to the CRB by the responsible investigator in accordance with Japanese regulations.

### 3.7 Data collection and management

Appropriate and authorized persons (investigators, clinical trial physicians, and clinical trial collaborators) will prepare a case report form (CRF) for each patient. All data recorded in the CRF must be consistent with the original material unless the data recorded directly in the CRF are used as the source material. According to the schedule presented in Table 1, the investigator will collect data at each patient visit during the study. The investigators will be provided access to an online, web-based, electronic data-capture system. Only the investigator will be able to enter and modify data in the electronic CRF (e-CRF). All study findings and documents will be regarded as confidential. Patients will be identified on the e-CRF by their patient number and/or birth date, not by name. The confidentiality of the documents that identify the patient must be maintained by the investigator so that the anonymity of the participants is ensured. During the study, a sponsor-investigator will perform regular site visits to review the protocol compliance, conduct source data verification, assess drug accountability and management, and ensure that the study is being conducted according to pertinent regulatory and protocol requirements.

### 3.8 Statistical analysis method

This study was designed to test the noninferiority of CT-P13 to the originator IFX. The non-inferiority margin was decided based on the results of 2 clinical studies^[19,20]^; the members of the present studys research group agreed with the validity of the studies design to assume the ’historical evidence of sensitivity to drug effects (HESDE) detailed in the U.S. Food and Drug Administration (FDA) guidance^[28]^ among these 2 studies and IFX-SIRIUS STUDY I (i.e., the present study) and to use them as a historical control for this study. Using the results of these 2 prior studies, we examined the proportions of patients with a clinical relapse during the observation period of 24 weeks among those who had achieved clinical remissions, at the time of enrollment, with TNF inhibitors. The difference between these 2 studies is in the treatment administered during the observation period. For the patients in the 1 study,^[20]^ the treatment with TNF inhibitors was continued during the observation period; in the other study,^[19]^ these medications were stopped during the observation period. The inferiority margin, 11.2%, was decided as the lower boundary of the 95% confidence interval of the difference between these 2 proportions. Regarding the expected proportion of patients with a clinical relapse in the IFX-SIRIUS STUDY I, we assume that it will be approximately the same as the results of study,^[20]^ which was16.0%.

The null hypothesis for noninferiority is rejected when the upper boundary of the confidence interval of the difference in the proportion of patients with experience of a clinical relapse during the study period is not larger than the value of 27.2% (i.e., the summation of the noninferiority margin, 11.2%, and the expectation of the proportion of patients with experience of a clinical relapse during the study period, 16.0%). All of the confidence intervals of proportions will be obtained using the Wilson score interval.

The analysis from which the main results are obtained is that on the Per Protocol Set—which consists of the patients evaluated for the primary endpoint in (at least) 2 scheduled visits; 1 at baseline and the other at the visit at week 24 or at which a clinical relapse is observed—in order to maintain the validity with the assumption of the HESDE^[28]^ with patients in the 2 historical control studies.

Other statistical analyses are planned to be exploratively conducted on the relationships between results obtained as the exploratory outcomes, which are detailed above in Section 3.5.3.

## 4 Discussion

The introduction of biologics into clinical practice has dramatically improved the management of a number of immune-mediated inflammatory diseases, including RA.^[29]^ However, the currently available biologics are expensive, which has led to restricted treatment access for patients with RA.^[30,31]^ Biosimilars offer cost-savings and health gains for patients and will play an important role in treating rheumatic diseases.^[9,31,32]^ A drug's price is one of the key factors for drug selection, and the cost-saving for CT-P13 was 48%(in terms of average NHI price as of February, 2020) compared with originator IFX in Japan. Since the introduction of biosimilars could reduce financial burdens on healthcare budgets, the Japanese government is promoting the use of biosimilars. However, some physicians are cautious about the application of biosimilars in clinical settings because they have questions about the efficacy and safety of biosimilars. The use of biosimilars will be promoted if patients on stable treatment with an originator biologic can safely be switched to the biosimilar.

The efficacy and safety equivalence of CT-P13 to originator IFX in patients with RA has been demonstrated.^[8,33,34]^ It was also reported that switching from originator IFX to CT-P13 is not inferior to continued treatment with originator IFX.^[9,10]^ However, it is difficult to produce generic drugs that have the same molecular structure as the originator biologic because biologics are high-molecular-weight compounds, and thus biosimilars are fundamentally different from generic drugs. It will therefore be most important to establish pharmacovigilance databases across countries that are adequate to monitor the biosimilars efficacy and safety after marketing approval.

The principal objective of the study is to evaluate whether switching from originator infliximab to CT-P13 is not inferior in maintaining a nonclinical relapse to continued treatment with originator IFX in RA patients achieving clinical remission. The strength of this study is its prospective evaluation of therapeutic efficacy using not only clinical disease activity indices but also MSUS to accurately and objectively evaluate disease activity at the joint level in a patient series drawn from multiple centers with a standardized evaluation by MSUS. We will also comprehensively analyze the serum levels of many biomarkers such as cytokines and chemokines. We will explore whether parameters at baseline can be used to predict non-relapse after switching from originator IFX to CT-P13 by integrating multilateral assessments (i.e., the clinical disease activity indices, MSUS findings, and biomarkers). We are also planning to evaluate the non-relapse status after the discontinuation of CT-P13 in patients who have not experienced a clinical relapse in the IFX-SIRIUS STUDY II following the IFX-SIRIUS STUDY I. It would be cost-effective if we could predict from the results of these 2 consecutive studies that patients on stable treatment with originator IFX could switch to CT-P13 without a relapse and could then discontinue CT-P13 without a relapse before switching.

## 5 Trial status

The IFX-SIRIUS I study received ethical approval on September 17, 2019. Recruitment started in December 2019 and is expected to finish June 30, 2022.

## Acknowledgments

We thank our colleagues and staff at the Rheumatology Department of Nagasaki University Hospital for their support.

## Author contributions

All authors have given their final approval of the manuscript to be published as presented.

**Conceptualization:** S. Kawashiri, T. Shimizu, H. Yamamoto, A. Kawakami

**Formal analysis:** S. Kawashiri, T. Shimizu, S. Sato, S. Morimoto

**Investigation:** S. Kawashiri, T. Shimizu, R. Sumiyoshi, A. Kawakami

**Methodology:** S. Kawashiri, T. Shimizu, S. Sato, S. Morimoto, N. Hosogaya, C. Fukushima, H. Yamamoto, A. Kawakami

**Project administration:** S. Kawashiri, T. Shimizu, Y. Kawazoe

**Writing – original draft:** S. Kawashiri, T. Shimizu

**Writing – review & editing:** S. Kawashiri, T. Shimizu, S. Sato, S. Morimoto, Y. Kawazoe, A. Kawakami.
